# Development of a Risk Matrix for Assessing PFAS in Food Packaging

**DOI:** 10.3390/foods15071183

**Published:** 2026-04-01

**Authors:** Katja Wack, Silvia Apprich, Johannes Bergmair, Manfred Tacker

**Affiliations:** 1 Circular Analytics TK GmbH, Canovagasse 7/14, 1010 Vienna, Austria; katja.wack@circularanalytics.com; 2 Department Applied Life Sciences, University of Applied Sciences, Favoritenstraße 226, 1100 Vienna, Austria; silvia.apprich@hcw.ac.at; 3 World Packaging Organization, Canovagasse 7/14, 1010 Vienna, Austria; j.bergmair@worldpackaging.org

**Keywords:** PFAS, PPWR, packaging, substances of concern, food contact materials, circular economy, PPWR compliance, risk matrix

## Abstract

The minimisation of substances of concern in packaging is a key objective of the European Union’s Packaging and Packaging Waste Regulation (PPWR), complementing existing legislation governing the safety of food contact materials. Per- and polyfluoroalkyl substances (PFAS) present particular challenges due to their persistence, chemical diversity, and documented use in certain food contact materials. Article 5 of the PPWR requires packaging to be designed and manufactured to minimise such substances throughout the life cycle. This study develops a structured, material-based PFAS risk matrix to support compliance screening for food packaging under Article 5. The approach combines scientific evidence on PFAS occurrence, functional applications, and analytical detection with material classification systems used in recyclability assessments. Packaging materials are categorised by their likelihood of PFAS relevance, enabling proportionate prioritisation of efforts. Application of the matrix shows that fibre-based materials with grease- or water-resistant treatments exhibit higher relevance than glass, untreated paper, or polyethylene terephthalate (PET). The framework also clarifies the role of total fluorine (TF) and extractable organic fluorine (EOF) as supportive, material-specific indicators rather than standalone compliance metrics. By integrating PFAS considerations into design, sourcing, and portfolio management, the framework promotes proactive chemical risk governance aligned with circular economy objectives.

## 1. Introduction

Food packaging plays a critical role in protecting food quality and safety by preventing contamination, extending shelf life, and enabling efficient storage and distribution. At the same time, packaging represents a continuous interface between industrial materials and consumers, making it a relevant pathway for chemical exposure. In recent years, per- and polyfluoroalkyl substances (PFAS) have emerged as a prominent concern in this context due to their documented use in specific packaging applications, high persistence, and increasing regulatory scrutiny [1,2].

Per- and polyfluoroalkyl substances (PFAS) comprise a large class of fluorinated organic compounds characterised by highly stable carbon–fluorine bonds that confer exceptional chemical and thermal stability and environmental persistence. Structurally, PFAS consist of a fluorinated carbon chain combined with a polar functional group (e.g., carboxylate or sulfonate), resulting in amphiphilic properties that enable surface activity such as oil and water repellency. These characteristics underpin their historical use in certain food contact materials, particularly in grease-resistant paper and fibre-based packaging [3].

PFAS have historically been used in specific food packaging applications where demanding barrier performance is required, particularly in grease- and water-resistant fibre-based materials such as fast-food wrappers, microwave popcorn bags, and moulded fibre trays. In plastic packaging, PFAS are more commonly associated with fluoropolymer-based processing aids used during polymer extrusion rather than with intentional surface treatments. A more detailed overview of typical application areas, functional drivers, and substitution challenges is provided in Section 3.1.

Toxicological and environmental behaviour is strongly influenced by structure–activity relationships, particularly fluorinated chain length and functional groups. Long-chain PFAS such as PFOA and PFOS show higher bioaccumulation potential and longer biological half-lives, while shorter-chain PFAS are generally more mobile and persistent in environmental systems. These properties contribute to widespread environmental occurrence and potential dietary exposure through food and packaging contact materials [4].

From a regulatory perspective, food contact materials in the European Union are primarily governed by legislation aimed at protecting consumer health, while broader sustainability and circularity aspects of packaging are increasingly addressed through the Packaging and Packaging Waste Regulation (PPWR) [5]. Article 5 of the PPWR introduces a horizontal obligation to minimise substances of concern throughout the packaging life cycle, complemented by specific concentration limits for PFAS in food-contact packaging, creating new expectations for chemical risk management that extend beyond use-phase food safety. The implications of this regulatory shift, including the redistribution of responsibility across the packaging value chain, are treated in detail in the discussion chapter.

Against this background, the central challenge addressed in this paper is how to translate complex and heterogeneous scientific knowledge on PFAS in food packaging into a practical, material-based framework that supports compliance with Article 5 of the PPWR. This paper proposes an integrated PFAS risk matrix that links scientific evidence, packaging material composition, and regulatory requirements in order to enable proportionate, life-cycle-oriented risk classification. By focusing on Article 5 PPWR compliance risk rather than toxicological risk quantification, the proposed approach aims to support economic operators in prioritising minimisation efforts, documenting due diligence, and aligning food packaging design with circular economy objectives.

## 2. Materials and Methods

### 2.1. Literature Screening

A structured literature search was conducted using the Scopus database to establish the scientific evidence base for the development of the PFAS risk matrix. The following Boolean search string was applied to titles, abstracts, and keywords:

(“PFAS” OR “perfluoroalkyl” OR “polyfluoroalkyl”) AND (“packaging” OR “food contact material”)

The search yielded 371 records, including peer-reviewed journal articles, book chapters, and conference proceedings. After title and abstract screening, followed by full-text assessment based on predefined inclusion and exclusion criteria, 78 publications were retained for detailed qualitative evaluation.

Inclusion criteria comprised studies addressing PFAS occurrence, functional use, migration behaviour, or analytical determination in packaging materials intended for food contact. Publications focusing exclusively on environmental contamination unrelated to packaging applications or toxicological evaluation without material relevance were excluded. In addition to peer-reviewed literature, selected regulatory assessments, government reports, industry documentation, and patent literature were considered to capture documented monitoring data and regulatory perspectives relevant to EU compliance.

The objective of the literature screening was not to establish quantitative exposure levels or conduct a risk assessment in toxicological terms, but rather to identify recurring associations between PFAS and specific packaging materials, constituents, or functional uses. Particular attention was given to functional drivers such as grease resistance, water repellency, release properties, and the use of polymer processing aids. These recurring associations form the empirical basis for the qualitative probability scoring applied in the PFAS risk matrix.

Emphasis was placed on materials that recur across food packaging portfolios and that have been shown to exhibit distinct PFAS occurrence patterns, including fibre-based packaging, moulded fibre articles, plastics, multilayer structures, and coated substrates.

### 2.2. Material Disaggregation and Constituent Analysis

To avoid treating packaging materials as homogeneous entities, all materials identified in the literature were disaggregated into their relevant constituents and functional elements. This constituent-level analysis aimed to identify the underlying drivers of PFAS relevance and included, in particular, surface treatments and coatings, polymer processing aids, lamination and sealing adhesives, sealing compounds, and non-intentionally added substances (NIAS) introduced through recycled content.

This approach enables differentiation between PFAS presence that is functionally driven (e.g., surface treatments or processing aids) and PFAS occurrence that is incidental or contamination-related. By focusing on constituents rather than finished articles alone, the framework captures material-specific PFAS pathways that are relevant for compliance assessment under Article 5 of the PPWR.

### 2.3. Material Classification Framework

The German Mindeststandard für recyclinggerechte Verpackungen [6] was used as the structural backbone for material classification. The Mindeststandard represents the de facto reference framework for recyclability assessments in Germany and is embedded in widely used digital tools supporting packaging compliance and design decisions, such as Packaging Cockpit. Anchoring the PFAS risk matrix to this framework ensures operational relevance and facilitates integration into existing packaging assessment workflows.

However, the Mindeststandard does not explicitly differentiate all functional attributes relevant to PFAS occurrence, such as specific coating chemistries, grease-proof paper grades, recycled fibre or plastic content, or multilayer constructions. To address this limitation, additional PFAS-relevant modifiers were introduced.

It is acknowledged that harmonised European design-for-recycling standards are currently being developed by CEN for all packaging materials, including plastics (e.g., prEN 18120). These harmonised standards are expected to replace national approaches such as the Mindeststandard within the PPWR framework. Until these standards are fully implemented and operational, the Mindeststandard provides a pragmatic interim basis for material classification.

### 2.4. PFAS Risk Matrix Design

Based on the combined evidence from literature screening, constituent analysis, and material classification, a qualitative PFAS risk matrix was developed. Probability scores ranging from 1 (very low probability) to 4 (high probability) reflect the likelihood that a given packaging material or constituent is associated with PFAS relevance. The scores do not represent toxicological risk, but rather the probability of PFAS presence or regulatory relevance in the context of Article 5 of the PPWR.

To avoid conceptual ambiguity, a distinction is made between risk matrix, risk classes, and risk assessment within the framework developed in this study. The risk matrix refers to the methodological tool that combines scientific evidence on PFAS occurrence, material composition, and functional attributes to derive probability-based classifications. The risk classes represent the categorical outcomes of this matrix and express the qualitative likelihood that a packaging material or constituent may be associated with PFAS relevance in the context of Article 5 of the PPWR. In contrast, risk assessment describes the overall evaluation process applied to a specific packaging system, in which individual components are analyzed using the matrix and aggregated to determine the overall PFAS compliance relevance of the packaging article. This distinction ensures that the matrix functions as a structured screening instrument, while the resulting risk classes serve as transparent decision-support indicators within a broader compliance assessment process.

**Figure 1:**
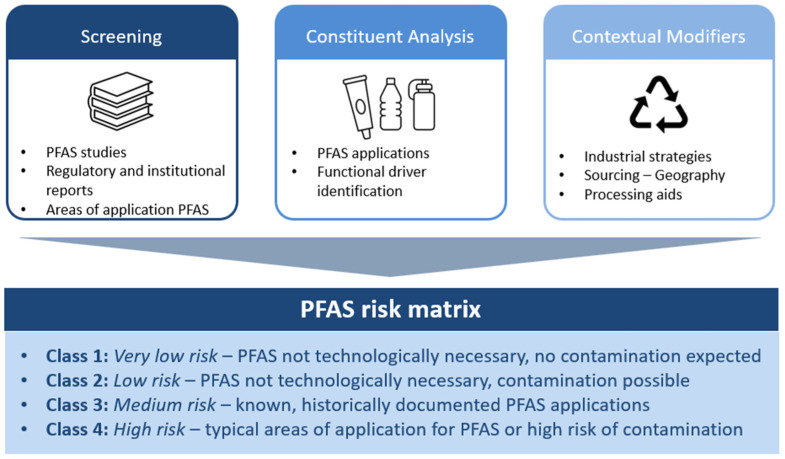
Conceptual framework for the development of the PFAS risk matrix for food packaging, linking scientific evidence, material classification, and compliance-oriented probability assessment.

Because certain PFAS-relevant drivers are not fully captured by base material categories alone, the matrix incorporates PFAS-relevant modifiers that allow classification by both base material (e.g., paper, plastic) and functional attributes that significantly influence PFAS probability. The combined evidence from literature, constituent analysis, and material classification was translated into qualitative probability scores for individual packaging constituents (see Figure 1).

In practical application, each packaging article is first assigned to the relevant material category as defined in the Mindeststandard. The corresponding PFAS probability score is then applied as a baseline classification. For composite and multilayer packaging, the highest probability score among the constituent materials is used as a conservative default, consistent with the precautionary logic underpinning Article 5 of the PPWR. Based on this approach, packaging articles are grouped into four PFAS risk classes ranging from very low to high probability of PFAS relevance.

### 2.5. Integration of Analytical Testing

Total fluorine (TF) and extractable organic fluorine (EOF) measurements were incorporated as supportive analytical screening tools within the framework. TF represents the sum of organic and inorganic fluorine present in a material, whereas EOF represents the fraction of extractable organic fluorine associated with PFAS and related substances.

These parameters are used for contextual screening and interpretation of PFAS relevance and do not replace the binding PFAS concentration limits or documentation requirements established under Article 5(5) of the PPWR. The interpretability of TF and EOF differs substantially between material classes, with stronger relevance for fibre-based materials than for plastics. Consequently, analytical results are interpreted in conjunction with material composition, functional use, and matrix-based risk classification rather than as standalone compliance indicators.

## 3. Results

### 3.1. PFAS in Food Contact Materials: Scientific Evidence

The scientific evidence on PFAS in food contact materials can broadly be grouped into three complementary domains: (i) occurrence studies documenting the presence of PFAS in packaging materials and finished food-contact articles, (ii) migration studies investigating the transfer of PFAS from packaging into food or food simulants, and (iii) analytical and methodological studies addressing detection approaches and analytical challenges. Together, these bodies of literature provide the empirical basis for identifying packaging materials and functional applications that exhibit recurring associations with PFAS. The following sections summarize the key findings from these domains with a focus on packaging materials relevant to the PFAS risk matrix developed in this study.

A substantial and growing body of scientific literature, supported by regulatory assessments and government surveys, documents the presence of PFAS in certain food contact materials (FCMs), including paper and board, plastics, and coated metals. Comprehensive reviews, regulatory mapping exercises, and large-scale monitoring studies consistently identify PFAS as one of the most prominent classes of substances of concern in food packaging, reflecting both historical uses and, in some cases, ongoing applications [1,2,7,8,9,10]. Early government-led inventories already highlighted food packaging as a key application area for highly fluorinated substances, alongside textiles and firefighting foams, underscoring the long-standing relevance of this exposure pathway [2,7].

Typical PFAS identified in food contact materials include fluorotelomer alcohols (FTOHs), fluorotelomer sulfonates (FTS), and polyfluoroalkyl phosphate esters (PAPs and diPAPs), which are commonly associated with grease-resistant paper and board treatments [11,12]. In addition, perfluoroalkyl carboxylic acids (PFCA) such as PFOA, PFHxA, PFPeA and PFBA are frequently detected as impurities, degradation products, or transformation products of precursor substances [10]. Reported concentrations vary depending on the packaging material and functional treatment. Fibre-based packaging historically shows the highest occurrence due to surface-applied treatments, whereas plastic materials typically exhibit lower concentrations related to processing aids or residual impurities [10,13]. Studies analysing paper and fibre-based food packaging have reported concentrations ranging from several ng/g to several hundred ng/g for individual PFAS compounds, particularly in grease-resistant applications [11,12]. By contrast, PFAS detected in plastic materials are generally associated with polymer processing aids or residual impurities and typically occur at lower concentrations [10,13]. These material-dependent occurrence patterns provide an important empirical basis for the material-oriented risk classification applied in the PFAS risk matrix developed in this study.

Among FCMs, paper and board have been identified as particularly PFAS-relevant due to their frequent treatment with grease- and water-repellent coatings. Early analytical investigations demonstrated the use of polyfluorinated surfactants and fluorotelomer-based coatings in certain paper and board food packaging [14]. Patent literature documents the application of fluorochemical treatments to cellulose substrates to impart grease resistance in food packaging laminates and paperboard structures [15]. The OECD’s assessment of PFAS use in paper and paperboard food packaging confirms that fluorinated substances have historically been applied to achieve oil and moisture resistance, particularly in fast food and takeaway applications such as wraps, boxes, cups, and microwave popcorn bags [8]. More recent scientific and regulatory evaluations further demonstrate that these materials exhibit a high potential for PFAS migration, especially when in contact with fatty foods or under elevated temperature conditions [1,16,17]. Migration experiments using standardised food simulants have repeatedly confirmed PFAS transfer from treated paper and board under realistic use conditions. [14,16]. Parallel to these findings, multiple research and development initiatives addressing PFAS substitution in paper-based food packaging have been reported in the literature and publicly available project documentation [17,18,19].

In contrast, PFAS detected in plastic FCMs are rarely associated with deliberate surface treatments designed to impart grease or moisture resistance. Reported occurrences are instead primarily linked to the use of fluorinated polymer processing aids, surface fluorination processes, impurities in additives, or contamination introduced via recycling streams [20]. Typical PFAS-containing additives include fluorinated masterbatches used to reduce melt fracture and the so-called “shark skin” phenomenon in film manufacturing [21,22], as well as certain slip and anti-block additives [23]. Fluoropolymer-based processing aids have been described for polyolefin film extrusion to reduce melt fracture and improve surface quality in blown film production [24]). Reviews of PFAS use patterns indicate that these substances are typically present at low concentrations [7]. Table 1 summarises packaging applications in which PFAS presence has been documented in the peer-reviewed literature and selected regulatory or institutional reports.

Fluorinated monomers and surface-modifying agents have also been disclosed in coating formulations for food and beverage containers (e.g., [25]).

Analytical investigations across Europe and beyond have identified a large number of individual PFAS and PFAS-related substances in FCMs. Reviews of the available analytical literature report the detection of several dozen distinct PFAS, spanning long- and short-chain perfluoroalkyl acids, fluorotelomer alcohols, sulfonates, and a wide range of precursor substances [1,16]. Importantly, long-chain PFAS targeted by regulatory restrictions and voluntary phase-outs continue to be detected in certain packaging materials, indicating the persistence of legacy contamination, transformation of precursor compounds, and the influence of recycling pathways [2,7,17].

Government inventories and market overviews further suggest that the total number of PFAS placed on the global market likely exceeds several thousand substances, many of which are used at low concentrations and remain insufficiently characterised from a toxicological perspective [2,8]. This high substance diversity presents substantial challenges for comprehensive analytical characterisation and regulatory control.

In addition to intentionally added PFAS, FCMs frequently contain NIAS, including impurities, polymerisation by-products, degradation products, and contaminants introduced via recycling streams. NIAS reported in FCMs include impurities, polymerisation by-products, degradation products, and contaminants introduced via recycling streams, many of which are not covered by existing authorisation lists or substance-specific migration limits. Recent methodological studies demonstrate that conventional targeted PFAS analysis captures only a fraction of the total fluorinated substances present in FCMs, which has led to increasing use of sum-parameter approaches such as TF and EOF for screening purposes [26]. TF refers to the sum of organic and inorganic fluorine present in a material, whereas EOF represents the fraction of fluorine associated with extractable organic compounds, including PFAS and PFAS-related substances. In this study, TF and EOF are applied as supportive screening parameters and not as compliance metrics.

Analytical determination of PFAS in food contact materials remains methodologically challenging due to the large number of substances and the structural diversity within the PFAS family. Targeted analytical methods typically rely on liquid chromatography coupled with tandem mass spectrometry (LC-MS/MS), enabling quantification of selected PFAS such as perfluoroalkyl carboxylic acids (PFCAs), sulfonates (PFSAs), and precursor compounds including fluorotelomer alcohols and polyfluoroalkyl phosphate esters [10]. However, targeted analysis usually covers only a limited subset of the thousands of PFAS that may occur in packaging materials. To address this limitation, complementary screening approaches based on sum parameters, including total fluorine (TF) and extractable organic fluorine (EOF), have increasingly been applied to estimate the presence of unidentified or polymeric PFAS [26,27]. The interpretation of these parameters can be complex because fluorine may also originate from non-PFAS sources or non-extractable polymeric materials, and analytical results may vary depending on extraction methods and matrix characteristics. Consequently, recent methodological frameworks recommend combining targeted PFAS analysis with sum-parameter screening and contextual material assessment to obtain a more comprehensive understanding of PFAS presence in food contact materials [10,27].

Interlaboratory comparison studies reveal considerable variability in TF and EOF results, particularly for paper-based materials, highlighting the analytical uncertainty associated with complex PFAS mixtures and heterogeneous material matrices [26].

Glass and metal packaging are typically free from PFAS, with the exception of certain organic coatings used for aluminium and metal cans, where PFAS may be present as defoamers, surfactants, or wetting agents [28]. For metal packaging, PFAS-free coating alternatives for metal packaging have been reported in the technical literature [28]. Printing inks can also represent a source of PFAS contamination. Historically, PTFE (polytetrafluoroethylene) waxes and fluorinated wetting agents have been used in inks, coatings, and varnishes, particularly for scratch-resistant applications [29].

**Table 1:** Packaging applications in which PFAS contamination has been documented in the published literature.

Packaging Application (PFAS-Relevant)	Sources
Fast food and takeaway paper packaging	[1,3,8,17,30,31,32,33]
Paper-based takeaway containers and trays	[3,17,30,33]
Cardboard packaging for pizza and hot food	[1,2,16,17,30,31,33,34,35]
Paper-based baking and cooking packaging (popcorn, baking paper, muffin cases, etc.)	[3,8,16,31,33,34,36]
“Compostable” or “bio-based” paper packaging with coating	[3,8,16,17,33]
Moulded fibre packaging made from plant-based materials (bagasse, palm leaves, straw, wheat fibres)	[3,17,26,30,33]
Paper packaging for baked goods and confectionery (bakery, pastry, doughnut and snack bags)	[1,2,3,17,30,32,33]
Paper and cardboard packaging for dry goods and confectionery (cereal, spaghetti and confectionery boxes)	[8,31,33,37]
Meat, fish and cheese wrapping paper (butcher’s/delicatessen paper)	[36,37]
Paper-based beverage cups (coated)	[14]
Plastic bowls, trays, cups	[1,3,20,37]
Flexible plastic packaging in general (bags, tubular bags, films)	[1,3,20,37]
Coated cans (interior paints/coatings)	[10]
Lids with fluorinated interior coatings	[10]
Laminated or multi-layer films (e.g., butter packaging)	[1,3,20,36]
Beverage composite carton (e.g., milk cartons)	[36]

### 3.2. Regulatory Framework and Implications for PFAS Assessment

European Union FCM legislation is primarily designed to protect consumer health by ensuring that materials and articles intended to come into contact with food do not transfer their constituents to food at levels that could endanger human health or cause unacceptable changes in food composition. Compliance is typically demonstrated through substance authorisation, migration testing, and conformity assessment under Regulation (EC) No 1935/2004 and related implementing measures. While this framework has proven effective in controlling individual substances during the use phase, it is largely oriented toward short-term consumer exposure and does not comprehensively address cumulative exposure, complex chemical mixtures, or impacts arising at the end of a product’s life cycle [1,16]. As there is currently no EU-wide positive list for paper and board food contact materials, the German BfR Recommendation XXXVI on paper and board intended for food contact is frequently used as a reference. This recommendation also includes provisions addressing PFAS [38]

The PPWR introduces a fundamentally different regulatory logic. Article 5 establishes a horizontal obligation to minimise substances of concern in packaging throughout the entire life cycle, explicitly linking chemical composition to recyclability, material recovery, and circular economy objectives. In addition, Article 5(5) specifies quantitative PFAS concentration limits for food-contact packaging applicable from August 2026, including thresholds for individual PFAS, the sum of PFAS determined by targeted analysis, and a total fluorine threshold triggering documentation obligations (Table 2). These limits complement, rather than replace, the general minimisation requirement and reinforce the need for material-level PFAS control.

The comparatively low concentration limit for individual PFAS (25 ppb) reflects the recognition that low-molecular-weight PFAS can be present at concentrations well below levels detectable by bulk fluorine screening alone. Such substances may occur either as impurities associated with polymeric PFAS or as intentionally added constituents at low concentrations that remain masked in total fluorine measurements. The inclusion of substance-specific limits therefore addresses regulatory blind spots inherent in sum-parameter approaches and ensures that mobile PFAS are captured even where TF concentrations remain low [27].

Unlike FCM legislation, Article 5 of the PPWR does not rely on migration limits but on the intrinsic composition of the packaging material. Compliance responsibility is assigned to the economic operator placing the packaging on the market, creating a shift from supplier-centric compliance toward shared but non-delegable responsibility across the packaging value chain. Within this context, risk-based, composition-oriented assessment frameworks, such as the clustering and matrix approach proposed in this study, are well aligned with the sustainability objectives of the PPWR.

For the purposes of Article 5, the PPWR adopts a structural definition of PFAS that is aligned with the OECD definition [9,39], encompassing substances containing at least one fully fluorinated methyl or methylene carbon atom, subject to specific regulatory clarifications and exclusions. This alignment ensures consistency between scientific PFAS assessments and regulatory implementation.

### 3.3. PFAS Risk Classification and Application of the Matrix

Anchoring the PFAS risk matrix to the *Mindeststandard für recyclinggerechte Verpackungen* enables direct integration of PFAS considerations into existing packaging assessment workflows and avoids the creation of parallel evaluation systems. The resulting classification supports risk-based prioritisation by allowing economic operators to focus resources on materials with the highest likelihood of Article 5 non-compliance rather than applying uniform controls across all packaging.

Table 3 and Table 4 present the PFAS risk classification of packaging constituents for plastic packaging (including multilayer structures) and for paper and board, aluminium, and steel packaging, respectively. No PFAS risks were identified for glass packaging. For rigid plastic packaging, PFAS relevance is generally low. In contrast, flexible plastic packaging exhibits a higher probability of PFAS relevance, primarily due to the use of polymer processing aids such as melt flow modifiers and slip agents. These additives are commonly applied to achieve high production throughput and to prevent surface defects such as sharkskin effects.

The PFAS risk associated with flexible plastic packaging is therefore driven less by individual polymer constituents and more by packaging characteristics, including film thickness, multilayer design, and production technology. Thin films and multilayer applications are associated with a higher probability of PFAS occurrence, albeit typically at low concentrations. Accordingly, flexible mono- and multilayer films are classified with a medium PFAS probability score. Given that processing aids represent the dominant PFAS risk pathway for plastic packaging, supplier declarations must explicitly address the use and control of such substances across the entire supply chain.

The matrix further facilitates targeted supplier engagement by identifying where detailed chemical information is most critical. For higher-risk materials, suppliers should provide transparency on PFAS presence and function, information on available alternatives, and evidence of compliance with evolving EU chemical restrictions. This requirement is particularly relevant for imported packaging, where equivalent documentation must be obtained for materials produced outside the EU.

Finally, the PFAS risk matrix is intended as a living tool. As scientific knowledge, regulatory requirements, and available alternatives evolve, classifications and supporting documentation should be reviewed and updated. By translating complex scientific and regulatory information into a transparent and reproducible system, the matrix supports systematic minimisation of substances of concern, prioritisation of substitution efforts, and alignment of food safety, chemical policy, and circular economy objectives under the PPWR.

The practical application of the packaging risk assessment is illustrated using a coated paper tray with a plastic multilayer lid and a paper banderole. The assessment begins with an evaluation of each individual constituent of the packaging, taking into account both the material composition (see Table 5 for details) and the potential impact of contamination arising from the production process.

For the plastic-coated paper tray, all material constituents are classified as low or very low risk (risk classes 1 or 2). However, the production process involves the extrusion of a thin multilayer film, which is classified as risk class 3 and is therefore included in the assessment. Tray forming itself is not considered a relevant risk factor. Consequently, the overall PFAS risk of the tray is determined by the highest risk identified within the column, resulting in a risk class 3. The same approach is applied to the plastic multilayer lid. In this case, the component with the highest risk is the anti-fog coating, which is assigned a risk class 4 (high risk). The extrusion process is classified as risk class 3. As the highest value determines the overall risk, the PFAS risk of the lid is therefore risk class 4 (high risk). For the paper banderole, the assessed risk is also risk class 4, mainly due to the use of recycled paper. However, this classification has no regulatory consequence, as the banderole is not intended to come into contact with food, and PFAS limit values do not apply to non-food-contact materials.

## 4. Discussion

### 4.1. Interpretation of the PFAS Risk Matrix in the Context of PFAS Governance

This study proposes a PFAS risk matrix that translates heterogeneous scientific evidence on PFAS occurrence in food contact packaging into a material-based compliance screening tool aligned with Article 5 of the PPWR. It operationalises the PPWR requirement to minimise substances of concern across the packaging life cycle by distinguishing packaging materials according to their probability of PFAS relevance.

Application of the matrix reveals clear material-dependent patterns. Fibre-based packaging materials, particularly grease-resistant paper, moulded fibre products, and coated paperboard, consistently emerge as having the highest PFAS relevance. In contrast, materials such as glass, untreated paper, and PET show consistently low PFAS relevance. These findings underscore the importance of risk-based approaches rather than uniform controls across all packaging types.

From a regulatory perspective, these material-specific patterns reinforce the rationale for a proportional implementation of Article 5. By enabling prioritisation of substitution, documentation, and verification efforts where PFAS relevance is highest, the matrix supports a key principle of EU regulation and provides a transparent basis for compliance-related decision-making.

### 4.2. Contribution to Implementation of Article 5 of the PPWR

A central challenge associated with Article 5 of the PPWR is its open-textured formulation. While the obligation to minimise substances of concern is clearly established, the regulation does not prescribe how conformity should be demonstrated in practice. This creates uncertainty for economic operators and increases the risk of inconsistent interpretation and enforcement during early implementation phases.

In addition to the implementation challenges associated with its open-textured formulation, Article 5 of the PPWR is expected to have substantial implications for packaging materials that have historically relied on PFAS for specific performance functions. The introduction of concentration limits for PFAS in food contact packaging, combined with the general obligation to minimise substances of concern, will likely lead to the phase-out of many PFAS-based applications, particularly in fibre-based packaging where fluorochemical treatments have been used to achieve grease and water resistance. As a consequence, packaging manufacturers and converters are increasingly required to adopt alternative barrier technologies, such as polymer dispersion coatings, wax-based treatments, or mineral barrier layers.

These regulatory changes also affect international supply chains. Packaging materials imported into the European Union must comply with the same PPWR requirements as those produced within the EU. This may pose particular challenges for certain packaging types, especially fibre-based materials originating from regions where PFAS restrictions are less stringent or where legacy fluorochemical treatments remain in use. Economic operators placing such packaging on the EU market remain responsible for compliance, which increases the importance of supplier documentation, material transparency, and targeted verification of material composition.

The PFAS risk matrix addresses this gap by offering a structured, transparent, and reproducible approach to compliance screening. Anchoring the matrix to the German *Mindeststandard für recyclinggerechte Verpackungen* aligns PFAS risk management with existing packaging governance infrastructures and widely implemented digital tools used in recyclability assessments. This integration embeds chemical considerations into established packaging design and evaluation workflows, avoiding the creation of parallel compliance systems and supporting sustainability-oriented decision-making.

By focusing on material composition and functional use rather than migration behaviour, the matrix is well aligned with the intrinsic material-based logic of Article 5. It enables economic operators to demonstrate systematic minimisation of substances of concern at the portfolio level, rather than relying on ad hoc or reactive measures.

### 4.3. Alignment of Analytical Testing with Risk-Based Classification

The integration of analytical PFAS testing approaches within the matrix framework further strengthens its applicability. Analytical results are often difficult to interpret in isolation, particularly where precursor or polymeric PFAS are present. By embedding testing within a matrix-informed workflow, analytical data gain contextual meaning and can be directly linked to compliance decisions under the PPWR.

The interpretation of analytical PFAS data is further complicated by methodological and matrix-related uncertainties. Targeted analytical methods generally rely on LC-MS/MS and cover only a limited number of well-characterised PFAS, while many precursor substances, polymeric PFAS, or transformation products remain analytically challenging to detect or quantify [10,27]. In addition, packaging matrices such as fibre-based materials, multilayer plastics, and coated substrates can influence extraction efficiency and detection limits, resulting in variability between analytical laboratories and analytical methods [10,16]. Recent interlaboratory studies demonstrate that both TF and EOF measurements may exhibit considerable variability depending on sample preparation, extraction protocols, and instrumental methods [26].

Another challenge lies in distinguishing between intentionally added PFAS and background contamination originating from recycled materials, processing aids, or legacy substances present in production equipment or supply chains [7,10]. Such uncertainties complicate the interpretation of analytical results in isolation and may limit their usefulness as standalone compliance indicators.

In this context, risk-based pre-screening tools such as the PFAS risk matrix proposed in this study can support proportionate testing strategies by identifying packaging categories where analytical verification is most relevant. By combining material composition, functional attributes, and available analytical evidence, the matrix provides a structured framework for interpreting analytical results and prioritising further investigation where the probability of PFAS relevance is highest.

Although Article 5(5) introduces a TF threshold that may trigger documentation obligations for food-contact packaging, measurements of TF or EOF do not in themselves demonstrate compliance. Such results must be interpreted in conjunction with material composition, functional use, and targeted PFAS analysis.

Importantly, the relevance of fluorine-based screening parameters differs markedly between material classes. For paper and board, elevated TF and EOF values correlate strongly with PFAS presence, reflecting the frequent use of fluorinated surface treatments. In this context, fluorine-based screening can serve as an effective trigger for further investigation. In contrast, such correlations do not hold for plastic packaging, where PFAS, if present, are typically associated with polymer processing aids, surface treatments, or recycling-related contamination rather than bulk material composition. Consequently, TF or EOF values in plastics do not reliably indicate the presence or concentration of individual PFAS and are not suitable as standalone screening tools.

The tiered testing strategy supported by the matrix enables proportionate evidence generation, avoiding unnecessary testing for low-probability materials while ensuring adequate scrutiny of higher-probability applications. In this way, the matrix functions as an interpretative layer between analytical data and regulatory obligations.

### 4.4. Implications for the Packaging Supply Chain

Brand owners, retailers, and private-label operators exert substantial influence over chemical outcomes in packaging through their control of specifications, material selection, and supplier approval processes. Under the PPWR, this influence is translated into explicit legal responsibility, as the economic operator placing packaging on the market must ensure minimisation of substances of concern across the entire packaging life cycle.

Packaging manufacturers and converters are central actors in this governance framework, as they determine material formulation, coating application, lamination, and processing conditions. While manufacturers are expected to provide transparent and verifiable information on PFAS presence, functional necessity, and substitution options, compliance responsibility cannot be transferred upstream. Information provided by suppliers must be actively evaluated and contextualised by downstream economic operators.

Importers occupy a particularly sensitive position, as they place packaging or packaged goods from third countries on the EU market while often having limited influence over upstream chemical choices. Nevertheless, compliance obligations apply in full. Importers must therefore exercise due diligence equivalent to that of EU-based manufacturers and brand owners, including obtaining substance-related information where feasible and verifying alignment with EU chemical safety and sustainability objectives. Generic compliance statements or reliance on non-EU regulatory standards are insufficient.

The introduction of concentration limits for PFAS in food contact packaging under Article 5(5) is also expected to have broader implications for packaging design and material selection across the supply chain. Applications that historically relied on fluorochemical treatments, particularly grease- and water-resistant fiber-based packaging, may increasingly require substitution with alternative barrier technologies such as polymer dispersions, wax-based coatings, or mineral barrier layers. This transition may occur at different speeds across global markets. Consequently, packaging imported from regions where PFAS restrictions are less stringent may present an elevated compliance risk for economic operators placing such materials on the EU market. Risk-based screening tools can therefore play an important role in identifying packaging categories where targeted supplier engagement or analytical verification is most relevant.

Within this governance context, the PFAS risk matrix operationalises these obligations by providing a structured mechanism to prioritise supplier engagement, documentation depth, analytical verification, and substitution efforts. By embedding Article 5 considerations into packaging design, sourcing, and supplier qualification processes, the matrix supports preventive action and reduces reliance on corrective measures triggered by enforcement or market pressure.

### 4.5. Implications for Circular Economy and Recyclability

PFAS present a particular challenge at the interface of chemical safety and circular economy policy. Their persistence and resistance to degradation mean that even low concentrations can compromise recycling streams and contribute to long-term contamination of secondary materials. By explicitly incorporating end-of-life and recyclability considerations into the compliance risk concept, the matrix extends beyond traditional food safety perspectives.

The findings indicate that packaging materials with high PFAS relevance often coincide with problematic recyclability profiles, particularly for fibre-based materials incorporating surface treatments. In this respect, the matrix contributes to a more integrated understanding of packaging sustainability.

### 4.6. Limitations and Future Perspectives

Several limitations of the proposed framework should be acknowledged. Firstly, the matrix relies on qualitative probability scoring derived from literature evidence and functional use patterns rather than quantitative exposure or concentration data. While appropriate for Article 5 compliance screening, this limits its applicability for health risk assessment or regulatory limit-setting. Secondly, the scientific literature on PFAS in food packaging remains uneven across materials and regions and reported detection frequencies may partly reflect analytical focus rather than true absence of PFAS. As new data emerge, particularly for plastics, coatings, and recycled materials, matrix classifications may require updating.

Thirdly, design-for-recycling frameworks are currently undergoing regulatory transition. Although the German *Mindeststandard für recyclinggerechte Verpackungen* provides a practical and widely applied classification basis, it does not explicitly differentiate all PFAS-relevant functional attributes. Harmonised European standards currently under development by CEN (e.g., prEN 18120) are expected to replace national approaches within the PPWR framework. Until these standards are fully implemented, some loss of granularity is unavoidable.

Finally, enforcement practices under the PPWR are still evolving. The extent to which authorities will accept qualitative, risk-based documentation as evidence of minimisation may vary across Member States, particularly during early implementation phases.

Future research should focus on validating and refining the matrix through empirical application, including case studies across diverse packaging portfolios and supply chains. Greater harmonisation of PFAS analytical methods and clearer regulatory guidance on acceptable evidence for Article 5 compliance would further strengthen the framework. More broadly, the approach developed in this study may be transferable to other substance groups of concern, supporting a shift toward group-based, life-cycle-oriented chemical governance in packaging.

## 5. Conclusions

This study develops a structured, material-based PFAS risk matrix to support compliance with Article 5 of the PPWR in the context of food packaging. By integrating scientific evidence on PFAS occurrence in food contact materials with material classification frameworks commonly used in recyclability assessments, the proposed approach translates complex and heterogeneous chemical information into a practical and operational compliance-screening tool.

The results demonstrate that the relevance of PFAS in food packaging is strongly material-dependent. Fibre-based materials incorporating grease- or water-resistant treatments consistently exhibit higher compliance relevance than materials such as glass, untreated paper, or PET. These findings support a differentiated, risk-based implementation of Article 5, enabling economic operators to prioritise minimisation, substitution, and documentation efforts where they are most effective. While the matrix does not assess toxicological risk or consumer exposure, it provides a transparent and reproducible method for operationalising the preventive intent of the PPWR at the material and portfolio levels.

By embedding PFAS considerations into packaging design, sourcing decisions, supplier engagement, and portfolio-level reviews, the framework facilitates a shift from reactive compliance toward proactive chemical risk governance. The integration of tiered analytical testing within a matrix-based workflow further enhances proportionality and regulatory relevance, particularly in light of analytical uncertainty and evolving standardisation. TF and EOF measurements are positioned as supportive analytical tools within this framework and do not replace the binding PFAS concentration limits or documentation requirements established under Article 5(5) of the PPWR.

Overall, the proposed approach illustrates how life-cycle-oriented, risk-based classification can contribute to the systematic minimisation of substances of concern while supporting recyclability and circular economy objectives. As regulatory expectations under the PPWR and related EU chemical policies continue to evolve, such integrative frameworks provide a pragmatic pathway for aligning food packaging safety, sustainability, and governance in practice.

## Figures and Tables

**Table 2:** Maximum concentrations of PFAS in food contact packaging as stated in PPWR Art. 5(5)(a)–(c).

PPWR	Maximal Concentration of PFAS in Food Contact Packaging
Art. 5(5)(a)	25 ppb for any PFAS with targeted PFAS analysis (polymeric PFAS excluded from quantification)
Art. 5(5)(b)	250 ppb for the sum of PFAS measured as the sum of targeted PFAS analysis (polymeric PFAS excluded from quantification)
Art. 5(5)(c)	50 ppm for PFAS (including polymeric PFAS)

**Table 3:** Risk matrix for paper and board packaging: qualitative PFAS probability classification of paper-based packaging constituents based on material composition and functional attributes. Risk classes represent the qualitative likelihood of PFAS relevance (1 = very low probability; 2 = low probability; 3 = medium probability; 4 = high probability).

	Materials	Literature	Functional Drivers	Risk Class
**Paper**				
**Main body**	Virgin paper		Virgin cellulose fibres are not intrinsically associated with PFAS; occurrence mainly linked to surface treatments.	1
	Recycled paper	[8,17,20,30,32,33]	PFAS may be introduced via recycled fibre streams and legacy treated paper products.	4
	Fibre casting virgin	[17,26,30,33,40]	PFAS relevance increases where moulded fibre requires grease- or water-resistant barrier treatments.	3
	Fibre casting recycled material	[2,8,20,32,33]	Raw materials/recycling: PFAS may already be present in the source material, even without intentional use.	4
	Cellophane		Base material typically PFAS-free; risk may arise from applied coatings or seal layers.	1
	Starch moulded parts		Do not require fluorinated additives	1
**Paper treatment**	Dry strength agent PVOH		Polyvinyl alcohol acts as a dry-strength agent and is not fluorinated.	1
	Dry strength agent starch		Starch-based strength additives are not associated with PFAS chemistry.	1
	Dry strength agent other polymers		Conventional dry-strength additives are typically non-fluorinated.	1
	Wetting agent		Do not require fluorinated additives	1
	Impregnating agent (e.g., for increased grease resistance)	[8,10,16,17,20,30,32,34,35,36]	Classic PFAS application area (also for paper straws, for example)	4
	Mineral fillers	[26]	Inorganic fillers such as calcium carbonate are PFAS-free.	1
**Barriers and surface finishing of paper**	Metallisation		PFAS relevance mainly linked to primers or coatings rather than the metal layer itself.	1
	Polymer dispersion coating	[7,20]	Barrier coatings may contain additives providing grease or moisture resistance that contain PFAS	3
	Silicone coating	[1]	Low, except when additives are used for better wetting and for papers with additional grease resistance/easy clean properties.	2
	Paraffin, wax, oil	[1]	Conventional hydrophobic barriers typically do not involve PFAS.	1
**Additional layers**	AlOx, SiOx, metallisation		Inorganic barrier layers are PFAS-free; risk may arise from primers or adhesion promoters.	1
**Adhesive application in multi-layer construction**	Dispersion adhesive	[7]	Inorganic barrier layers are PFAS-free; risk may arise from primers or adhesion promoters.	2
	Hot melt adhesive		Hydrocarbon-based systems are PFAS-free.	1
	Starch-based adhesive		Natural polymer adhesive systems without fluorinated components.	1
**Composite materials**	Aluminium lamination		Increased risk with high-performance laminating adhesives/topcoats	2
	Plastic film lamination		PFAS possible in laminating adhesives, additives; protective coatings	3
**Decoration**	Direct printing	[7,33]	Printing Inks for paper packaging are typically free from PFAS.	1
	Foil embossing		Film embossing is often multi-layered—PFAS were used in release/separating layers.	3
	Varnish	[17]	Functional coatings may include fluorinated additives for surface performance.	3
**Label material**	Paper (not wet-strength)		See respective material	1
	Paper (wet-strength)		See respective material	2
	Plastic		See respective material	
**Label adhesives**	Dispersion	[7]	Fluorinated surfactants may be used in certain formulations.	2
	Hot melt	[7]	Conventional thermoplastic adhesives typically PFAS-free.	1
	Starch-based	[7]	Bio-based adhesive systems generally PFAS-free.	1
**Seam bonding** **Adhesives**	Dispersion	[7]	PFAS relevance possible depending on formulation.	2
	Hot-melt adhesive		Typically not associated with PFAS.	1
	Starch-based		Typically not associated with PFAS.	1
**Metal**				
**Aluminium**	Aluminium	[10]	Base metal does not contain PFAS.	1
	Inner coating	[1,3]	Typical area of application: CO2-containing beverages with a long shelf life.	3
	Outer coating	[10]	Functional coatings may contain fluorinated additives (abrasion-resistant, anti-fingerprint)	3
	Compound (in closures)	[7,34]	PFAS presence depends on sealing compound formulation. In closures (twist-off caps, crown caps, etc.). Classification 3 if the recipe is disclosed	3
**Steel**	Steel cans	[10]	Steel substrate is PFAS-free	1
	Inner coating	[1,3]	Food-contact coatings may include fluorinated additives. Typical area of application.	3
	Outer coating	[10]	In high-performance coatings (abrasion-resistant, anti-fingerprint)	3
	Compound (in closures)	[7,34]	PFAS presence depends on sealing compound formulation. In closures (twist-off caps, crown caps, etc.) Classification 3 if the recipe is disclosed	3

**Table 4:** Risk matrix for plastic and metal packaging: qualitative PFAS probability classification of constituents in plastic and metal packaging based on material composition and functional attributes. Risk classes represent the qualitative likelihood of PFAS relevance (1 = very low probability; 2 = low probability; 3 = medium probability; 4 = high probability).

	Materials	Literature	Additional Information	Risk Class
**Polymer Mono**	PET-A; PET-G; PET-C (amorphous, glycol-modified, and crystalline polyethylene-terephthalate) virgin		PFAS may enter the material during processing, such as extrusion, as contaminants from additives or processing aids, rather than being intentionally added to modify material properties	1
	rPET (recycled PET)	[1]	PFAS may enter the material during processing, such as extrusion, as contaminants from additives or processing aids, rather than being intentionally added to modify material properties	1
	HDPE (High-Density Polyethylene) virgin	[1,20,41]	PFAS may enter the material during processing, such as extrusion, as contaminants from additives or processing aids, rather than being intentionally added to modify material properties	1
	rHDPE (recycled HDPE)	[1,20]	not in food contact materials	2
	LDPE (Low-Density Polyethylene) virgin	[1,20]	PFAS may enter the material during processing, such as extrusion, as contaminants from additives or processing aids, rather than being intentionally added to modify material properties	1
	rLDPE (recycled LDPE)	[1,20]	not in food contact materials	2
	PP (Polypropylene) virgin	[1]	PFAS may enter the material during processing, such as extrusion, as contaminants from additives or processing aids, rather than being intentionally added to modify material properties	1
	rPP (recycled PP)	[1]	not in food contact materials	2
	PEF (Polyethylene furanoat) virgin		PFAS may enter the material during processing, such as extrusion, as contaminants from additives or processing aids, rather than being intentionally added to modify material properties	1
	PS (Polystyrene) virgin		PFAS may enter the material during processing, such as extrusion, as contaminants from additives or processing aids, rather than being intentionally added to modify material properties	1
	rPS (recycled PS)		not in food contact materials	2
	PBT (Polybutadiene terephthalate) virgin		PFAS may enter the material during processing, such as extrusion, as contaminants from additives or processing aids, rather than being intentionally added to modify material properties	1
**Polymer Multilayer**	EVOH layer (Ethylene vinyl alcohol-copolymer)	[1,30]	PFAS relevance depends mainly on adhesives or tie layers in multilayer structures.	2
	PVOH layer (Polyvinyl alcohol)	[1,30]	PFAS unlikely in the polymer itself; risk linked to coatings or additives.	3
	PE peel layer	[1,30]	Additives used to modify peel performance may introduce PFAS.	3
	PP peel layer	[1,30]	Additives used to modify peel performance may introduce PFAS.	3
	PVDC (Polyvinylidene dichloride)	[1,30]	Barrier polymer itself typically PFAS-free; additives may influence risk	2
	Lamination adhesive	[30]	PFAS may occur as surfactants or formulation additives.	3
**Coating/painting/vapor deposition**	Coating (not defined)		Unknown coating chemistry may include fluorinated additives.	4
	AlOx	[7,30]	Inorganic barrier layer PFAS-free; risk may arise from primer layers.	2
	Antifog coating	[7,30]	Typical application containing PFAS	4
	EVOH coating		Risk depends on formulation additives in dispersion coatings.	3
	PVOH coating	[7,30]	Risk depends on formulation additives in dispersion coatings.	3
	SiOx	[30]	Inorganic layer PFAS-free; primers may introduce PFAS.	2
	Metallisation	[30]	Metal layer PFAS-free; primers may introduce PFAS.	2
	Acrylate coating	[7,30]	Low risk, but fluorinated additives may be used to modify surface properties.	2
	Functional coating/varnish	[7,30]	High-performance coatings are typical sources of PFAS may contain fluorinated additives.	3
	Varnish	[30]	PFAS may occur where surface performance additives are used.	2
	Direct printing	[30]	PFAS historically used as wetting agents in printing inks.	2
**Adhesive**	Adhesive (not defined)		Unknown formulation may contain fluorinated additives.	4
	Acrylate adhesive	[7]	PFAS may occur as surfactants or processing additives.	2
	PU adhesive		Additives may introduce PFAS depending on chemistry.	3
	Hotmelt		Thermoplastic adhesives generally PFAS-free.	1
**Other**	Compound/sealing material	[7,34]	Sealing compounds may contain fluorinated additives depending on formulation.	3
	Absorbent layers—PE, PP, PS	[1,7]	PFAS may occur via additives or processing aids in multilayer absorbent structures.	3

**Table 5:** PFAS compliance risk assessment and hotspot identification for a composite food packaging system (paper/PE tray, multilayer plastic lid, printed paper sleeve). Risk classes represent the qualitative likelihood of PFAS relevance (1 = very low probability; 2 = low probability; 3 = medium probability; 4 = high probability).

Packaging Composition	Tray	Risk Class	Lid	Risk Class	Paper Sleeve (Printed)	Risk Assessment
Constitutent 1	PE	1	PE	1	paper (recycled)	4
Constitutent 2	cardboard (virgin)	1	tie layer (specified)	2	print	2
Constitutent 3	PE	1	EVOH	1	hot melt adhesive	1
Constitutent 4	tie layer (specified)	2	tie layer (specified)	2		
Constitutent 5	EVOH	1	PE	1		
Constitutent 6	tie layer (specified)	2	anti-fog coating	4		
Constituent 7	PE	1				
Production process 1	Tray formation	0	flexible film extrusion	3		
Production process 2	flexible film extrusion	3				
**Risk classification**	**PE/cardboard/tie layer/EVOH/tie layer/PE**	**3**	**PE/tie layer/EVOH/tie layer/PE**	**4**	**Paper (recycled)**	**4**

## Data Availability

The original contributions presented in this study are included in the article. Further inquiries can be directed to the corresponding author.

## Abbreviations

The following abbreviations are used in this manuscript:

AlOx	Aluminium Oxide
CEN	European Committee for Standardization
EOF	Extractable Organic Fluorine
EVOH	Ethylene Vinyl Alcohol Copolymer
FCM	Food contact material
FTS	Fluorteleomer Sulfonate
HDPE	High-Density Polyethylene
LDPE	Low-Density Polyethylene
NIAS	Non-Intentionally Added Substances
OECD	Organisation for Economic Co-operation and Development
PAP	Polyfluoroalkyl Phosphate Ester
PBT	Polybutylene Terephthalate
PEF	Polyethylene Furanoate
PET	Polyethylene Terephthalate
PFAS	Per- and Polyfluoroalkyl Substances
PFOA	Perfluorooctanoic Acid
PFOS	Perfluorooctane Sulfonate
PP	Polypropylene
PPWR	Packaging and Packaging Waste Regulation
PS	Polystyrene
PU	Polyurethane
PTFE	Polytetrafluoroethylene
PVOH	Polyvinyl Alcohol
PVDC	Polyvinylidene Chloride
SiOx	Silicon Oxide
TF	Total Fluorine
